# The Effects of Elevated Tropospheric Ozone on Carbon Fixation and Stable Isotopic Signatures of Durum Wheat Cultivars with Different Biomass and Yield Stability

**DOI:** 10.3390/plants11223185

**Published:** 2022-11-21

**Authors:** Limin Ma, Chong Chen, Lorenzo Cotrozzi, Chengcheng Bu, Jiahong Luo, Guodong Yao, Guangyao Chen, Weiwei Zhang, Cristina Nali, Giacomo Lorenzini

**Affiliations:** 1 State Key Laboratory of Pollution Control and Resources Reuse, College of Environmental Science and Engineering, Tongji University, Shanghai 200092, China; 2 Key Laboratory of Yangtze River Water Environment, Ministry of Education, Tongji University, Shanghai 200092, China; 3 Department of Agriculture, Food and Environment, University of Pisa, Via del Borghetto, 80, 56124 Pisa, Italy; 4 CIRSEC, Centre for Climate Change Impact, University of Pisa, Via del Borghetto, 80, 56124 Pisa, Italy

**Keywords:** air pollution, oxidative stress, yield, ecophysiological parameters, stable carbon isotope, stable nitrogen isotope

## Abstract

Tropospheric ozone (O_3_) enrichment caused by human activities can reduce important crop yields with huge economic loss and affect the global carbon cycle and climate change in the coming decades. In this study, two Italian cultivars of durum wheat (Claudio and Mongibello) were exposed to O_3_ (80 ppb, 5 h day^−1^ for 70 consecutive days), with the aim to investigate the changes in yield and biomass, ecophysiological traits, and stable carbon and nitrogen isotope values in plants, and to compare the stable isotope responses under environmental stressors. Both cultivars showed a relative O_3_ tolerance in terms of photosynthetic performance, but in cultivar Mongibello, O_3_ was detrimental to the grain yield and plant biomass. The δ^13^C values in the leaves of plants identified that the impact of O_3_ on CO_2_ fixation by RuBisCO was dominant. The δ^15^N value showed significant differences between treatments in both cultivars at seven days from the beginning of the exposure, which could be considered an early indicator of ozone pollution. Under increasingly frequent extreme climates globally, the relationships among stable isotope data, ecophysiological traits, and agronomic parameters could help breed future cultivars.

## 1. Introduction

Tropospheric ozone (O_3_) is a major secondary air pollutant [1]. Despite efforts to reduce the emission of O_3_ precursors (e.g., nitrogen oxides and volatile organic compounds), concentrations of this pollutant are still elevated in many areas worldwide and are expected to rise further due to both anthropogenic activities and climate change [2,3]. Background O_3_ instantaneous levels in the Northern Hemisphere are estimated to increase from 35–50 ppb to 42–84 ppb in 2100, depending on the seasonal and spatial variability (1 ppb = 1.96 µg m^−3^, at 25 °C and 101.325 kPa) [4,5,6]. This increase is expected in hot-spot regions such as East Asia and the Mediterranean [7,8,9], although uncertain or even opposite tendencies have been predicted in other regions [10,11,12].

Ozone can severely affect plants by entering through the open stomata and producing reactive oxygen species (ROS), which react with all biological macromolecules [13,14,15]. Although plants can detoxify ROS [16], O_3_-induced oxidative stress commonly alters physiological processes such as carbon assimilation by inducing partial stomatal closure or less efficient stomatal control, reducing foliar pigment content, impairing the electron transport chain, contracting the efficiency of Calvin Cycle enzymes like RuBisCO, lowering the availability of photosynthates for reallocation, and increasing respiration rates [12,17]. Consequently, crop yield and grain quality would decline. For example, yield loss due to O_3_ has been predicted to range from 3 to 4% for rice, from 3 to 5% for maize, from 6 to 16% for soybean and from 7 to 12% for wheat [18].

Wheat is one of the most important stable crops worldwide and one of the most O_3_-sensitive crops [19]. *Triticum durum* Desf. (durum wheat) is a major member of the genus Triticum, being largely used for its relatively high gluten content [20,21]. It is mainly grown in the Mediterranean area, the Northern Plains between the USA and Canada, and in the desert of southwest of the USA and Northern Mexico, Turkey, Syria, North Africa, and other Asian countries [22,23]. Although most studies have focused on O_3_ effects on common wheat (*T. aestivum* L.), durum wheat has also been reported as O_3_ sensitive (e.g., Chen et al. [24]), even if somewhat contrasting results have been highlighted depending on concentration and duration of O_3_ exposure, as well as on genotypic differences in O_3_ tolerance (e.g., Gerosa et al. [25]).

Breeding of higher grain yield in wheat (and other crops) has been frequently related to increased stomatal conductance (g_s_), which generally fosters higher photosynthetic rates and cools down plants. However, the higher g_s_ can also potentially increase cultivar O_3_-sensitivity, as they absorb higher amounts of the pollutant [17]. Although O_3_-induced effects on plants are commonly investigated by discrete gas exchange measurements [17,26], photosynthetic/transpiration responses to environmental stress conditions may be better elucidated through the analysis of the stable carbon (δ^13^C) and nitrogen (δ^15^N) isotope compositions [27,28]. The physiological mechanism underlying ^13^C enrichment in stressed plants is usually attributed to a decrease in g_s_ and an increase in water use efficiency [29]. The δ^15^N values in plant tissues are instead primarily associated with the isotopic composition of the nitrogen (N) sources (soil, precipitation, N_2_ fixation, fertilization) and forms (NH_4_^+^, NO_3_^-^, organic N). It could be further influenced by the N-fixing bacteria and mycorrhizal fungi [30], and the δ^15^N values will change during N uptake, translocation, assimilation, and reallocation within the plant [31]. Although all these processes are influenced by environmental conditions [29], only a few studies have reported the effects of O_3_ stress on plant δ^13^C and δ^15^N (e.g., Saurer et al. [27]). Since ^13^C and ^15^N contents vary with leaf age and plant development, studies investigating variations of these parameters in response to O_3_ should include leaves at different phenological stages [29].

In our previously published research [24], we investigated the responses of two Italian durum wheat cultivars, i.e., Claudio and Mongibello, exposed to chronic O_3_ exposure (80 ppb, 5 h day^−1^, for 70 consecutive days), reporting that Claudio showed a higher O_3_ tolerance than Mongibello, and characterizing the cultivar-specific phenolic profiles. The present work represents a follow-up aimed at investigating the relationships between the ecophysiological responses and the δ^13^C and δ^15^N variations and elucidating the effects of O_3_ pollution on biomass and yield parameters of the investigated durum wheat cultivars. Outcomes from the present study could be useful for breeding wheat cultivars with a high tolerance to environmental stress.

## 2. Results and Discussion

### 2.1. Biomass and Yield Variations Induced by Ozone

Variations of biomass and yield parameters induced by O_3_ are shown in Table 1. The interaction cultivar (*Cv*) *× O_3_* was significant for total aboveground biomass, ear and grain dry weight (DW), thousand-grain weight, and the number of grains and spikelets per ear. A significant *O_3_* effect was reported on leaf and stem DW and the number of grains per spikelet. No significant effects were reported on the number of ears per plant. Total aboveground biomass, ear and grain DW, and thousand-grain weight were higher in controls of Mongibello than in Claudio (+32, +49, +54 and +51%, respectively). In Claudio and even more in Mongibello, O_3_ decreased total aboveground biomass (−30 and −40%, respectively), ear DW (−29 and −40%, respectively), and the number of grains per ear (−29 and −40%). Only in Mongibello, O_3_ also significantly decreased grain DW, thousand-grain weight, and the number of spikelets per ear (−38, −37 and −15%, respectively). Similarly, between cultivars, O_3_ reduced leaf and stem DW and the number of grains per spikelet (–32, –37 and –28%, as average, respectively).

The different behavior of these two cultivars in relation to O_3_ exposure showed that Mongibello was more sensitive, while Claudio was more resistant. Claudio showed a reduction in the ear DW but not in grain DW, although there was a decrease in the number of grains per ear. Therefore, Claudio produced ears with less but larger caryopsis, and the thousand-grain weight under O_3_ was also not significantly different from that in controls. Moreover, only in Mongibello O_3_ was detrimental to the most important agronomic parameter, the grain yield. This may be due to the greater sensitivity between inflorescence emission and anthesis during the critic period and decreased photosynthetic activity in Mongibello [32]. Another possible reason is that the photosynthetic pigments may not be exploited as antioxidants, and the delay in the activation of the xanthophyll de-epoxidation cycle has greater photoinhibition-related damages [33,34,35]. Similar conclusions were also carried out by Pleijel et al. [19], who reported that O_3_ (35.6 ± 10.6 ppb) had significant negative effects on grain yield (−8%), grain mass (−4%), harvest index (−2%), total aboveground biomass (−5%), starch concentration (−3%), starch yield (−11%), and protein yield (−6%) of 19 wheat cultivars, compared with charcoal filtered air (13.7 ± 8.8 ppb of O_3_). A more quantitative relationship between O_3_ concentrations and wheat yields was observed by Harmens et al. [15], who reported that wheat yield and thousand-grain weight declined linearly with increasing phytotoxic O_3_ dose above a flux threshold of Y (PODY). However, the wheat in different regions worldwide showed variant responses to O_3_. Pleijel et al. [36] compared the influence of O_3_ on the grain yield, average grain mass, and harvest index of wheat in Europe, Asia, and North America. They found that North American wheat was less sensitive than European and Asian ones, which responded similarly. The variation in responses across all three continents was smallest for the harvest index, followed by grain mass and yield.

### 2.2. Leaf Area (La) and Ecophysiological Parameters

The effects of *Cv*, *O_3_*, growth stage (*Gs*), and their interactions on leaf area (La), CO_2_ assimilation rate (A), g_s_, and maximum RuBP-saturated rate of carboxylation (V_cmax_) are reported in Table 2. No significant *Cv* effect and *Cv × O_3_ × Gs* interaction were found for these four indicators, whereas significant *O_3_ × Gs* interactions were reported on La and A. Meanwhile, significant *Cv × Gs* interactions were observed on La, g_s_, and V_cmax_, and a significant *Cv × O_3_* interaction was reported on A. The singular *O_3_* and *Gs* effects showed significant effects on all of the four indicators.

**Figure 1:**
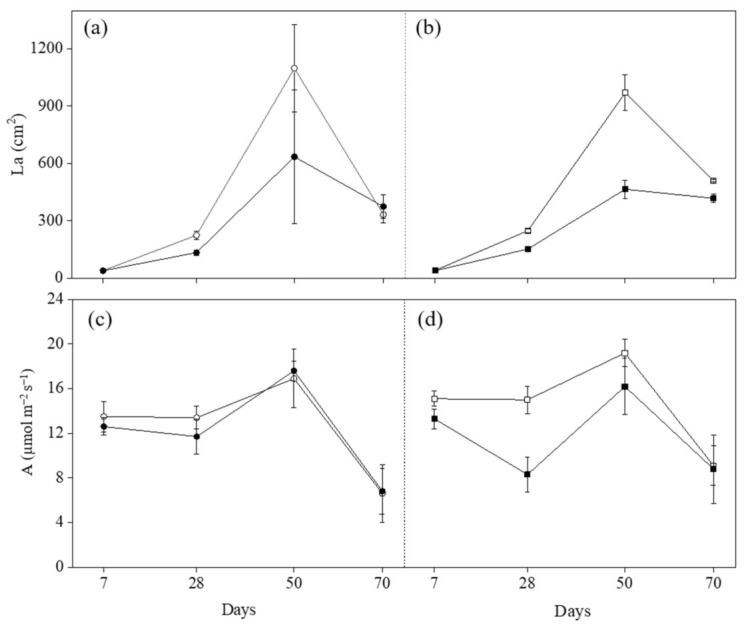
Leaf area (La) (**a**,**b**) and CO_2_ assimilation rate (A) (**c**,**d**) in durum wheat cultivars Claudio (circle, *left*) and Mongibello (square, *right*) exposed to 0 (open) or 80 (closed) ppb of ozone (5 h day^−1^) at different growth stages (7, 28, 50, 70 days from the beginning of exposure). Data are shown as mean ± standard deviation (n = 3). A vertical dashed line separates the cultivars.

**Figure 2:**
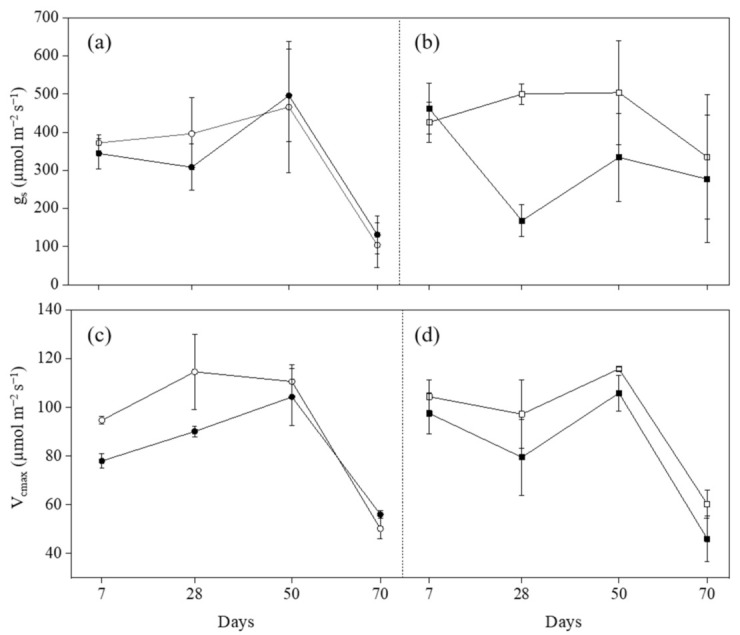
Stomatal conductance (g_s_) (**a**,**b**) and maximum RuBP-saturated rate of carboxylation (V_cmax_) (**c**,**d**) in durum wheat cultivars Claudio (circle, *left*) and Mongibello (square, *right*) exposed to 0 (open) or 80 (closed) ppb of ozone (5 h day^−1^) at different growth stages (7, 28, 50, 70 days from the beginning of exposure). Data are shown as mean ± standard deviation (n = 3). A vertical dashed line separates the cultivars.

Variations of La and A induced by *O_3_* are shown in Figure 1, and variations of g_s_ and V_cmax_ are shown in Figure 2. Ozone significantly decreased La at 50 days from the beginning of exposure (FBE) in both cultivars (−42% in Claudio and −52% in Mongibello; Table 3). The differences in La between Claudio and Mongibello at all growth stages were not significant in controls or exposed to O_3_ (Table 3). The La in both cultivars increased at 28 and 50 days FBE and decreased at 70 days FBE due to the senescence of plants. A significant reduction of A was observed at 28 and 50 days FBE in Mongibello exposed to O_3_ (−45% and −16%, respectively; Table 3), and A was lower at 70 days FBE in both cultivars compared with the other three growth stages [24]. Similarly to A, O_3_ decreased g_s_ at 28 and 50 days FBE in Mongibello (−66% and −34%, respectively; Table 3). The g_s_ in Claudio decreased at 70 days FBE compared with the previous growth stage in both groups (controls and O_3_-treated groups), while the difference in Mongibello was only observed in controls (Table 3). The significant differences between treatments were shown at 7 and 28 days FBE in Claudio and 28 days FBE in Mongibello for V_cmax_ (Table 3). In Claudio, the V_cmax_ in O_3_-treated plants was 18% and 21% lower than that in controls at 7 and 28 days FBE, respectively, and in Mongibello, the V_cmax_ in O_3_-treated plants was 18% lower than that in controls at 28 days FBE. The V_cmax_ decreased at 70 days FBE in both treatments for both cultivars.

**Figure 3:**
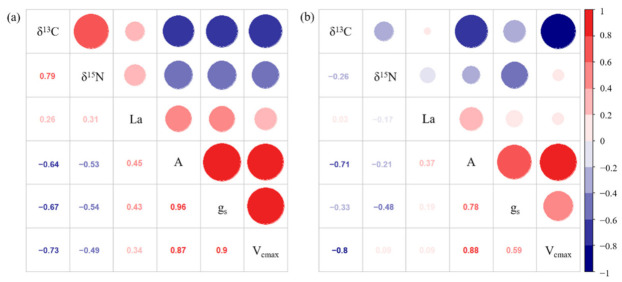
Pearson’s correlation matrix describing relationships among δ^13^C, δ^15^N, leaf area (La), CO_2_ assimilation rate (A), stomatal conductance (g_s_), and maximum RuBP-saturated rate of carboxylation (V_cmax_) in durum wheat cultivars Claudio (**a**) and Mongibello (**b**) exposed to 0 or 80 ppb of ozone (5 h day^−1^) at different growth stages (7, 28, 50, 70 days from the beginning of exposure).

Notably, positive correlations were found among the ecophysiological traits in both Claudio and Mongibello (Figure 3). The correlation coefficients for A with g_s_ and V_cmax_ were 0.96 and 0.87 in Claudio, and 0.78 and 0.88 in Mongibello, while that for g_s_ with V_cmax_ were 0.90 and 0.59 in Claudio and Mongibello, respectively. The correlation coefficients for La with A, g_s_, and V_cmax_ were relatively small, and they were 0.45, 0.43, and 0.34 in Claudio and 0.37, 0.19, and 0.09 in Mongibello.

La, A, g_s_, and V_cmax_ are all widely used photo-oxidative stress markers [37]. In this study, the reductions of La in both cultivars were only observed at 50 days FBE, and the reductions of A and g_s_ were only observed in Mongibello at 28 and 50 days FBE. The reductions of V_cmax_ were shown at 7 and 28 days FBE in Claudio and 28 days FBE in Mongibello. All these traits later recovered the same levels of control. These results indicate a relative O_3_ tolerance of durum wheat in photosynthetic performance, and the photosynthetic performance between the two cultivars was similar. Similar conclusions were reached in previous studies on other cultivars of wheat [24,25,38,39]. However, the temporary decrease of photosynthetic activity during the critic period (at 7, 28, and 50 days FBE) was demonstrated, and as mentioned above, this may be an important reason for the O_3_-induced reductions of some biomass and yield parameters in wheat cultivars.

### 2.3. δ^13^C Values

**Figure 4:**
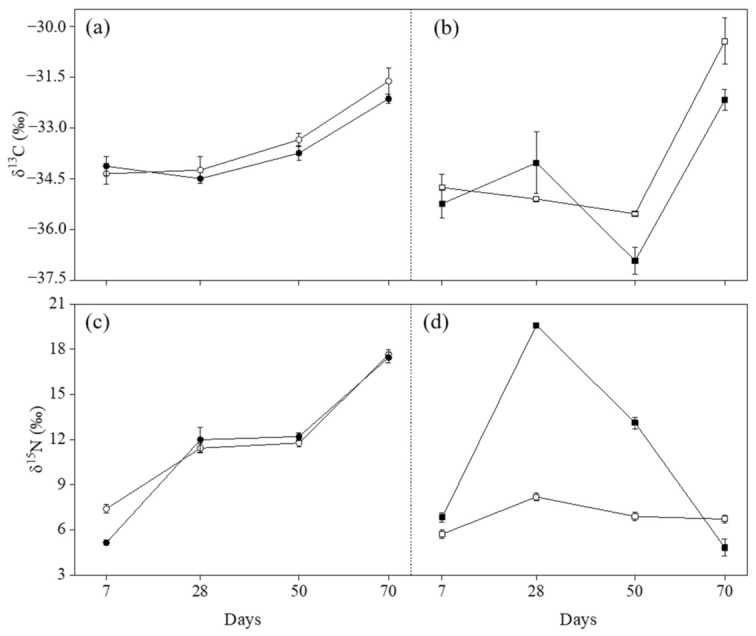
δ^13^C (**a**,**b**) and δ^15^N (**c**,**d**) values in durum wheat cultivars Claudio (circle, *left*) and Mongibello (square, *right*) exposed to 0 (open) or 80 (closed) ppb of ozone (5 h day^−1^) at different growth stages (7, 28, 50, 70 days from the beginning of exposure). Data are shown as mean ± standard deviation (n = 3). A vertical dashed line separates the cultivars.

Ozone induced an increase of δ^13^C values at 28 days FBE (+1.070‰) and a significant decrease of δ^13^C values at 50 and 70 days FBE in Mongibello (−1.387‰ and −1.739‰, respectively; Figure 4b; Table 3), while the δ^13^C differences between treatments in Claudio at all of the growth stages and in Mongibello at seven days FBE were not significant (Figure 4a,b; Table 3). The δ^13^C values changed gently at 7, 28, and 50 days FBE in both cultivars, but the values increased significantly at 70 days FBE with the senescence of plants (Figure 4a,b). Significant *Cv × O_3_ × Gs* interactions were found for both δ^13^C and δ^15^N (actually, only the *Cv × O_3_* effect on δ^13^C was not significant; Table 4).

^12^CO_2_ is preferred to plants compared with ^13^CO_2_ during photosynthetic CO_2_ assimilation, and both CO_2_ diffusion across the stomata and CO_2_ fixation by RuBisCO could contribute to the discrimination of ^13^CO_2_ in favor of ^12^CO_2_ in C_3_ plants [27,40]. The metabolic capacity decreases with the senescence of plants, and the δ^13^C values in the plants had an upward trend through time, especially at 70 days FBE (Figure 4a,b). The δ^13^C value in the air (δ^13^C_air_) is about −8‰, and that in the C_3_ plants (δ^13^C_plant_) ranges from −36‰ to −22‰ [41,42], the plant is isotopically lighter than atmospheric CO_2_, and their relationship could be quantitatively described using the following equation [27,43,44].

*δ^13^C_plant_* = *δ^13^C_air_*−*a*−(*b*−*a*)(*p_i_*/*p_a_*) (1)

where *a* is the fractionation occurring due to diffusion, and the value is about +4.4‰; *b* is the net fractionation caused by carboxylation (mainly by RuBisCO), and the value is about +27‰; *p_i_* is the partial pressure of CO_2_ in the mesophyll; *p_a_* is the partial pressure of CO_2_ in ambient air.

As is shown in Equation (1), when the g_s_ limits photosynthesis, the *p_i_/p_a_* value is relatively low, leading to a relatively small fractionation and less δ^13^C negative values compared to controls [44]. The other situation is that when the impact on CO_2_ fixation by RuBisCO is dominant, the increase in *p_i_/p_a_* value will result in larger fractionation and more negative δ^13^C values [43]. Therefore, the carbon isotopic composition of plants is a good indicator to show how some environmental conditions (e.g., air pollutants, water availability, temperature) affect the uptake and fixation of CO_2_. In this study, A and g_s_ at 28 and 50 days FBE in Mongibello decreased significantly, and these traits returned to control values (Figure 1d and Figure 2b). V_cmax_ showed significant reductions only at 28 days FBE (Figure 2d). However, the grain yield and biomass of plants still decreased significantly (Table 1). The δ^13^C values could better explain this phenomenon under the long-term accumulated O_3_ exposure condition, and the impact of O_3_ on CO_2_ fixation by RuBisCO was dominant. In other words, the process of CO_2_ fixation by RuBisCO is more sensitive to O_3_ than the stomatal conductance in Mongibello. Correlation analysis further verified this conclusion (Figure 3). In Mongibello, the correlation coefficients for δ^13^C with V_cmax_ and A were –0.80 and –0.71, respectively, while that for δ^13^C with the stomatal conductance was only –0.33. In Claudio, the correlation for δ^13^C with V_cmax_ (–0.73) was stronger than with g_s_ (–0.67), and the correlation coefficient for δ^13^C with A was –0.64. Therefore, the δ^13^C values well explained why the temporary decrease of photosynthetic activity during the critic period (at 28 and 50 days FBE) affected the O_3_-induced reductions of some yield parameters.

### 2.4. δ^15^N Values

In Claudio, O_3_ only induced a significant reduction of δ^15^N values at seven days FBE (−2.252‰; Table 3), and the δ^15^N values increased through time in controls and O_3_-treated plants, especially at 28 and 70 days FBE (Figure 4c). In Mongibello, significant differences in δ^15^N values between treatments were observed at all of the growth stages (Table 3). O_3_ increased the δ^15^N values at 7, 28, and 50 days FBE (+1.105‰, +11.402‰, and +6.224‰, respectively) and decreased the values at 70 days FBE (−1.895‰). The δ^15^N values in both treatments increased at 28 days FBE and then decreased at 50 and 70 days FBE, which was especially noticeable in O_3_-treated plants (Figure 4d).

The δ^15^N value is also a commonly applicable ecotoxicological indicator of O_3_ pollution early on, as significant differences between treatments were observed in both cultivars at seven days FBE (Table 3). Claudio recovered later, while O_3_-treated Mongibello showed a significant increase in the δ^15^N values at 7, 28, and 50 days FBE compared with the control treatment (Figure 4c,d; Table 3). Previous studies proved the most significant changes in the isotope ratios in the non-protein nitrogen fraction, followed by the soluble protein and the structural protein fractions [45,46], and this enrichment could be explained by accelerated N metabolism. Furthermore, wheat leaves exposed to O_3_ increased their permeability to soluble substances such as amino acids and proteins before the visible injury, caused by membrane alterations [47]. Such a leakage led to the enrichment of the proportion of N-containing substances with larger δ^15^N values according to general isotope effects [48]. In addition, plants react to O_3_ stress by narrowing their stomatal openings at the following growth stages, N fixation is changed from the air more towards the soil, which has higher δ^15^N values compared with N in the air [49]. The correlation coefficient of –0.48 for δ^15^N with the g_s_ in Mongibello could verify this inference (Figure 3). It should also be noted that O_3_ exposure was not the only reason causing the stable isotope responses. Other stresses, such as heat and drought, might give similar responses to those shown in this study. Therefore, more information is needed to determine wheat’s characteristic stable isotope responses of wheat under different stress conditions.

The stable isotope data in plants is a good indicator and recorder of historical environmental information. Under increased frequent extreme climates globally [50], the δ^13^C and δ^15^N values in wheat can tell humans what kind of climatic conditions they have encountered, and the relationships among stable isotope data, ecophysiological traits, and agronomic parameters could help screen more tolerant crops quickly and efficiently in the early stage and eventually benefit breeding future cultivars.

## 3. Materials and Methods

### 3.1. Plant Material and Ozone Exposure

Details of plant material and O_3_ exposure used in the experimental activity are already reported by Chen et al. [24]. In short, seeds of durum wheat cultivars Claudio and Mongibello were sown in plastic pots, which were then maintained in a greenhouse under charcoal-filtered air until the stage of “first leaf unfolded/second leaves unfolded” (BBCH-Code [BBCH-C] 11–12, i.e., 20-day-old seedlings) [51]. Uniform-sized seedlings were then distributed among four had hoc built perspex fumigation chambers (595 × 540 × 1975 mm; two chambers per treatment; twelve seedings per chamber (three plants for each collection period)) and exposed to charcoal-filtered air (controls, assumed as 0 ppb of O_3_) or a target concentration of 80 ± 10 ppb of O_3_ (ca. 160 µg m^−3^ at 20 °C and 101.3 kPa, 5 h day^−1^, in the form of a square wave between 10:00 and 15:00 h) for 70 consecutive days. For further details about fumigation facilities, plant management, and environmental conditions, see Landi et al. [52] and Chen et al. [24].

Ecophysiological analyses and leaf (the third fully expanded mature leaves) collections for δ^13^C and δ^15^N investigations of control and O_3_-treated plants were performed at seven (BBCH-C 13), 28 (BBCH-C 23–32), 50 (BBCH-C 59) and 70 (BBCH-C 65–69) days FBE. For each combination of cultivar, O3 treatment, and time, two completely expanded leaves were collected, mixed, divided into aliquots, instantly frozen in liquid nitrogen, and stored at −80 °C until δ^13^C and δ^15^N analyses. At the end of the exposure, biomass and yield parameters were also assessed (see Section 3.4).

### 3.2. Leaf Area (La) and Ecophysiological Analyses

La was measured using a Leaf Area Meter (YMJ-G, Fengtu, Shandong, China). Ecophysiological analyses were carried out between 11:00 and 13:00 h (i.e., when maximum gas exchanges usually occur) on one completely expanded leaf per plant [24]. The A and the V_cmax_ were measured under light-saturated conditions (photosynthetic active radiation of about 1200 μmol photons m^−2^ s^−1^), ambient CO_2_ concentration (ca. 390 μmol mol^−1^) and 60% of relative humidity, using an Infrared Gas Analyzer (CIRAS-1; PP-Systems, Hitchin, Hertfordshire, UK). The acclimation time for the leaves before the measurements were 40 min. One fully expanded mature leaf per plant and three plants per treatment were chosen for the measurements. Stomatal conductance was measured using a Stomatal Conductance Meter (FS-3080C, Fangsheng, Heibei, China).

### 3.3. δ^13^C and δ^15^N

The C isotopic composition of a sample is expressed on the δ^13^C scale, defined as Equation (2).

(2) δC13=C13/C12sample−C13/C12standardC13/C12standard×1000‰

where *^13^C/^12^C* is the ratio of the number of ^13^*C* atoms to the number of ^12^*C* atoms in the sample or standard. Vienna Pee Dee Belemnite (V-PDB) was the standard, defined as 0‰ [53].

The N isotopic composition of a sample is expressed on the δ^15^N scale, defined as Equation (3).

(3) δN15=N15/N14sample−N15/N14standardN15/N14standard×1000‰

where *^15^N/^14^N* is the ratio of the number of ^15^*N* atoms to the number of ^14^*N* atoms in the sample or standard. The atmospheric air was used as the standard, defined as 0‰ [54].

For each combination of cultivar, O_3_ treatment, and time, 400 μg of leaves were collected and wrapped in a tin foil cup. Triplicates of δ^13^C and δ^15^N analyses of each sample were performed by an Element Analyzer-Isotope Ratio Mass Spectrometer (EA-IRMS, Thermo Fisher, Waltham, MA, USA). The EA was set as follows: the helium pressure (99.999%) was 250 kPa, and the flow rate was 100 mL min^−1^. The reference flow rate of the Thermal Conductivity Detector was 80 mL/min^−1^, and the temperature of the Oxidation Furnace and Reduction Furnace was 960 °C and 680 °C, respectively. The chromatographic column was a packed column whose temperature was 55 °C. The pressure of oxygen (99.999%) was 300 kPa, and the flow rate was 180 mL min^−1^. The oxygen injection time was 1 s. The IRMS was operated at an accelerating potential of 10 kV. Ions were generated by an electron impact of 70 eV. The emitted energy for δ^13^C analysis was 1.5 mA, and it was 2.0 mA for δ^15^N analysis.

The reference materials for the calibration of ^13^C were caffeine (IAEA-600), carbamide (UREA), and L-glutamic acid (USGS 41). Their stable carbon isotope ratios relative to V-PDB were −1.17‰, −48.63‰, and 37.626‰, respectively. The reference materials for calibration of ^15^N were caffeine (IAEA-600), potassium nitrate (IAEA-NO-3), carbamide (UREA), and L-glutamic acid (USGS 41). Their stable nitrogen isotope ratios relative to atmospheric air were 37.83‰, 4.70‰, −0.30‰, and 47.6‰, respectively. All reference materials were from Sigma-Aldrich (Shanghai, China).

### 3.4. Determinations of Biomass and Yield Parameters

The leaves, stems, ears, and grains of the durum wheat in fully ripe and developed condition were placed in an oven at 103 °C for 24 h and weighed to calculate the biomass (g DW). The biomass sum of the leaves, stems, and ears was calculated to obtain the total above-ground biomass (g DW). The number (n) of ears plant^−1^, grains ear^−1^, spikelets ear^−1^, and grains spikelet^−1^ were obtained by manual counting. Thousand-grain weight (g) of the durum wheat was given by:

(4) $$\mathrm{Thousand}\;\mathrm{grain}\;\mathrm{weight}\;\left(g\right)=\frac{{\mathrm{Grain}\;\mathrm{weight}\;\mathrm{plant}}^{-1}\left(g\;\mathrm{DW}\right)}{{\mathrm{Ears}\;\mathrm{plant}}^{-1}\left(n\right)\times {\mathrm{Grains}\;\mathrm{ear}}^{-1}\left(n\right)}\times 1000$$

### 3.5. Statistics

The normal distribution of data was preliminary analyzed following the Shapiro–Wilk test. The effects of the cultivar (*Cv*), growth stage (*Gs*), O_3_ exposure (*O_3_*), and their interactions on ecophysiological traits and δ^13^C and δ^15^N values were tested using a three-way analysis of variance (ANOVA). The effects of *Cv* and *O_3_* and their interaction on biomass and yield parameters investigated at the end of the exposure were tested using a two-way ANOVA. The Tukey HSD test was used as the *post hoc* test. Relations among ecophysiological traits and δ^13^C and δ^15^N values were evaluated using Pearson’s correlations. Effects with *p* ≤ 0.05 were considered statistically significant. Statistical analyses were carried out in SPSS version 25 (IBM, New York, NY, USA).

## 4. Conclusions

The tropospheric O_3_ enrichment caused by human activities in the coming decades can reduce the yields of important crops with a huge economic loss worldwide. Though O_3_ is unlikely to be the only stress during plants’ growth and development, the variation of stable carbon (δ^13^C) and nitrogen (δ^15^N) isotope values in the leaves is a useful tool to understand the response of the carbon sequestration mechanism of plants, and it was also found to be a potential marker for the ecophysiological traits of plants at all the stages of growth. In this study, the durum wheat cultivar Mongibello showed a significant reduction in grain yield and plant biomass, and the cultivar Claudio showed a relative O_3_ tolerance. The δ^13^C values explained the mechanism of the O_3_-induced reductions of some yield parameters, and the δ^15^N values played an important role in the early indication of oxidative stress.

## Figures and Tables

**Table 1:** Biomass and yield parameters in wheat cultivars Claudio and Mongibello exposed to 0 (control) or 80 ppb of ozone (O_3_, 5 h day^−1^) for 70 consecutive days.

	Claudio		Mongibello		*p*		
	Control	O_3_	Control	O_3_	*Cv*	*O_3_*	*Cv* × *O_3_*
					(*df*: 1)	(*df*: 1)	(*df*: 1)
Leaf biomass (g DW plant^−1^)	2.1 ± 1.3	1.5 ± 0.4	2.3 ± 1.0	1.5 ± 0.4	0.833	**<0.001**	0.833
Stem biomass (g DW plant^−1^)	3.5 ± 1.2	2.4 ± 0.7	4.3 ± 1.4	2.5 ± 0.7	0.523	**<0.001**	0.542
Total aboveground biomass (g DW plant^−1^)	10.11 ± 4.11 b	7.1 ± 2.3 a	13.3 ± 5.1 c	8.0 ± 2.4 a	**<0.001**	**<0.001**	**0.015**
Ears plant^−1^ (n)	3.7 ± 1.3	4.0 ± 1.3	4.2 ± 1.1	3.7 ± 1.1	0.888	0.888	0.577
Ear weight (g DW plant^−1^)	4.5 ± 1.6 b	3.2 ± 1.2 a	6.7 ± 2.6 c	4.0 ± 1.3 ab	**<0.001**	**<0.001**	**0.014**
Grain weight (g DW plant^−1^)	2.4 ± 1.1 a	2.2 ± 1.0 a	3.7 ± 0.9 b	2.3 ± 0.6 a	**0.018**	**0.007**	**0.026**
Thousand grain weight (g)	24.7 ± 3.0 a	20.3 ± 1.4 a	37.4 ± 3.5 b	23.4 ± 1.4 a	**<0.001**	**<0.001**	**0.024**
Grains ear^−1^ (n)	42.0 ± 9.0 c	30.0 ± 6.1 b	40.0 ± 7.9 c	24.0 ± 9.0 a	**0.003**	**<0.001**	**0.013**
Spikelets ear^−1^ (n)	16.4 ± 2.3 b	15.5 ± 1.9 b	16.0 ± 1.8 b	13.6 ± 2.0 a	**0.018**	**<0.001**	**0.025**
Grains spikelet^−1^ (n)	2.8 ± 0.6	2.0 ± 0.6	2.5 ± 0.6	1.8 ± 0.9	0.536	**<0.001**	0.551

Note: Data are shown as mean ± standard deviation. *p*-values of two-way ANOVA for the effects of the cultivar (*Cv*), ozone exposure (*O_3_*), and their interactions on yield parameters of wheat are shown. Significant values (*p* ≤ 0.05) are shown in bold. According to the Tukey HSD *post ho*c test, different letters in each row indicate significant differences (*p* ≤ 0.05). Abbreviations: *df*, degrees of freedom; DW, dry weight.

**Table 2:** *p*-values of three-way ANOVA for the effects of the cultivar (*Cv*; Claudio and Mongibello), growth stage (*Gs*; 7, 28, 50 and 70 days), ozone treatment (*O_3_*; 0 and 80 ppb, 5 h day^−1^) and their interactions on leaf area (La), CO_2_ assimilation rate (A), stomatal conductance (g_s_) and maximum RuBP-saturated rate of carboxylation (V_cmax_) of durum wheat.

	*df*	La	A	g_s_	V_cmax_
*Cv*	1	0.894	0.160	0.103	0.687
*O_3_*	1	**<0.001**	**0.002**	**0.017**	**<0.001**
*Gs*	3	**<0.001**	**<0.001**	**<0.001**	**<0.001**
*Cv* × *O_3_*	1	0.478	**0.019**	0.053	0.724
*Cv* × *Gs*	3	**0.050**	0.195	**0.021**	**0.003**
*O_3_* × *Gs*	3	**<0.001**	**0.046**	0.057	0.120
*Cv* × *O_3_* × *Gs*	3	0.866	0.351	0.258	0.160

Note: Significant values (*p* ≤ 0.05) are shown in bold. Abbreviation: *df*, degrees of freedom.

**Table 3:** Three-way ANOVA analysis describing significant differences among leaf area (La), CO_2_ assimilation rate (A), stomatal conductance (g_s_), maximum RuBP-saturated rate of carboxylation (V_cmax_), δ^15^N and δ^13^C in durum wheat cultivars Claudio (CLAU) and Mongibello (MONG) exposed to 0 (CTR) or 80 (O_3_) ppb of ozone (5 h day^−1^) at different growth stages (7, 28, 50, 70 days from the beginning of exposure).

				*p* Values					
				La	A	g_s_	V_cmax_	δ^15^N	δ^13^C
CTR	7 days	CLAU	MONG	0.991	0.278	0.515	0.178	**<0.001**	0.216
	28 days	CLAU	MONG	0.790	0.278	0.214	**0.019**	**<0.001**	**0.012**
	50 days	CLAU	MONG	0.159	0.122	0.646	0.466	**<0.001**	**<0.001**
	70 days	CLAU	MONG	0.055	0.094	**0.008**	0.161	**<0.001**	**0.001**
O_3_	7 days	CLAU	MONG	1.000	0.632	0.160	**0.009**	**<0.001**	**0.002**
	28 days	CLAU	MONG	0.833	**0.025**	0.097	0.142	**<0.001**	0.154
	50 days	CLAU	MONG	0.067	0.341	0.057	0.822	**0.004**	**<0.001**
	70 days	CLAU	MONG	0.634	0.177	0.084	0.165	**<0.001**	0.907
CLAU	7 days	CTR	O_3_	0.991	0.539	0.735	**0.024**	**<0.001**	0.487
	28 days	CTR	O_3_	0.317	0.249	0.291	**0.001**	0.067	0.431
	50 days	CTR	O_3_	**<0.001**	0.632	0.717	0.378	0.155	0.226
	70 days	CTR	O_3_	0.642	0.891	0.744	0.416	0.530	0.116
MONG	7 days	CTR	O_3_	0.982	0.223	0.663	0.335	**0.001**	0.147
	28 days	CTR	O_3_	0.292	**<0.001**	**<0.001**	**0.017**	**<0.001**	**0.002**
	50 days	CTR	O_3_	**<0.001**	**0.047**	**0.046**	0.169	**<0.001**	**<0.001**
	70 days	CTR	O_3_	0.307	0.837	0.484	0.051	**<0.001**	**<0.001**
CLAU	CTR	7 days	28 days	**0.047**	0.945	0.772	**0.008**	**<0.001**	0.727
		7 days	50 days	**<0.001**	**0.025**	0.260	**0.031**	**<0.001**	**0.004**
		7 days	70 days	**0.003**	**<0.001**	**0.003**	**<0.001**	**<0.001**	**<0.001**
		28 days	50 days	**<0.001**	**0.022**	0.399	0.574	0.259	**0.009**
		28 days	70 days	0.237	**<0.001**	**0.001**	**<0.001**	**<0.001**	**<0.001**
		50 days	70 days	**<0.001**	**<0.001**	**<0.001**	**<0.001**	**<0.001**	**<0.001**
	O_3_	7 days	28 days	0.297	0.539	0.663	0.095	**<0.001**	0.259
		7 days	50 days	**<0.001**	**0.002**	0.073	**0.001**	**<0.001**	0.238
		7 days	70 days	**0.001**	**<0.001**	**0.014**	**0.004**	**<0.001**	**<0.001**
		28 days	50 days	**<0.001**	**<0.001**	**0.029**	0.052	0.483	**0.025**
		28 days	70 days	**0.011**	**0.002**	**0.038**	**<0.001**	**<0.001**	**<0.001**
		50 days	70 days	**0.006**	**<0.001**	**<0.001**	**<0.001**	**<0.001**	**<0.001**
MONG	CTR	7 days	28 days	**0.027**	0.945	0.373	0.314	**<0.001**	0.298
		7 days	50 days	**<0.001**	**0.008**	0.348	0.115	**<0.001**	**0.022**
		7 days	70 days	**<0.001**	**<0.001**	0.275	**<0.001**	**0.002**	**<0.001**
		28 days	50 days	**<0.001**	**0.007**	0.961	**0.013**	**<0.001**	0.184
		28 days	70 days	**0.006**	**<0.001**	0.053	**<0.001**	**<0.001**	**<0.001**
		50 days	70 days	**<0.001**	**<0.001**	**0.047**	**<0.001**	0.564	**<0.001**
	O_3_	7 days	28 days	0.212	**0.002**	**0.001**	**0.016**	**<0.001**	**0.001**
		7 days	50 days	**<0.001**	0.054	0.128	0.242	**<0.001**	**<0.001**
		7 days	70 days	**<0.001**	**0.004**	**0.031**	**<0.001**	**<0.001**	**<0.001**
		28 days	50 days	**0.001**	**<0.001**	0.051	**0.001**	**<0.001**	**<0.001**
		28 days	70 days	**0.006**	0.732	0.193	**<0.001**	**<0.001**	**<0.001**
		50 days	70 days	0.596	**<0.001**	0.492	**<0.001**	**<0.001**	**<0.001**

Note: Significant values (*p* ≤ 0.05) are shown in bold.

**Table 4:** *p*-values of three-way ANOVA for the effects of the cultivar (*Cv*; Claudio and Mongibello), growth stage (*Gs*; 7, 28, 50 and 70 days), ozone treatment (*O_3_*; 0 and 80 ppb, 5 h day^−1^) and their interactions on δ^13^C and δ^15^N values of durum wheat.

	*df*	δ^13^C	δ^15^N
*Cv*	1	**<0.001**	**<0.001**
*O_3_*	1	**0.001**	**<0.001**
*Gs*	3	**<0.001**	**<0.001**
*Cv ×O_3_*	1	0.090	**<0.001**
*Cv × Gs*	3	**<0.001**	**<0.001**
*O_3_ × Gs*	3	**<0.001**	**<0.001**
*Cv ×O_3_ × Gs*	3	**0.001**	**<0.001**

Note: Significant values (*p* ≤ 0.05) are shown in bold. Abbreviation: *df*, degrees of freedom.

## Data Availability

The data presented in this study are available on request from the corresponding authors.
